# The Emerging Roles of Nanoparticles in Managing the Environmental Stressors in Horticulture Crops—A Review

**DOI:** 10.3390/plants14142192

**Published:** 2025-07-15

**Authors:** Mohamed K. Abou El-Nasr, Karim M. Hassan, Basma T. Abd-Elhalim, Dmitry E. Kucher, Nazih Y. Rebouh, Assiya Ansabayeva, Mostafa Abdelkader, Mahmoud A. A. Ali, Mohamed A. Nasser

**Affiliations:** 1 Horticulture Department, Faculty of Agriculture, Ain Shams University, Cairo 11241, Egypt; 2 Department of Agricultural Microbiology, Faculty of Agriculture, Ain Shams University, Cairo 11241, Egypt; 3 Department of Environmental Management, Institute of Environmental Engineering, RUDN University, Moscow 117198, Russia; 4 Department of Agronomy, Faculty of Agricultural Sciences, Akhmet Baitursynuly Kostanay Regional University, Kostanay 110000, Kazakhstan; 5 Horticulture Department, Faculty of Agriculture, Sohag University, Sohag 82524, Egypt

**Keywords:** biotic, abiotic stresses, salt, drought, heavy metals, nanomaterials, sustainability

## Abstract

The primary worldwide variables limiting plant development and agricultural output are the ever-present threat that environmental stressors such as salt (may trigger osmotic stress plus ions toxicity, which impact on growth and yield of the plants), drought (provokes water stress, resulting in lowering photosynthesis process and growth rate), heavy metals (induced toxicity, hindering physiological processes also lowering crop quantity and quality), and pathogens (induce diseases that may significantly affect plant health beside productivity). This review explores the integrated effects of these stressors on plant productivity and growth rate, emphasizing how each stressor exceptionally plays a role in physiological responses. Owing to developments in technology that outclass traditional breeding methods and genetic engineering techniques, powerful alleviation strategies are vital. New findings have demonstrated the remarkable role of nanoparticles in regulating responses to these environmental stressors. In this review, we summarize the roles and various applications of nanomaterials in regulating abiotic and biotic stress responses. This review discusses and explores the relationship between various types of nanoparticles (metal, carbon-based, and biogenic) and their impact on plant physiology. Furthermore, we assess how nanoparticle technology may play a role in practices of sustainable agriculture by reducing the amount of compounds used, providing them with a larger surface area, highly efficient mass transfer abilities, and controlled, targeted delivery of lower nutrient or pesticide amounts. A review of data from several published studies leads to the conclusion that nanoparticles may act as a synergistic effect, which can effectively increase plant stress tolerance and their nutritional role.

## 1. Introduction

Recently, horticulture crops have suffered deterioration and production losses due to adverse environmental conditions [1,2,3,4]. Environmental stressors, including biotic and abiotic stresses, are identified as any external factors that negatively affect plant growth and development [5,6]. Water shortage is the primary source of drought and salinity, which is indirectly leading to heavy metal toxicity in contemporary agriculture. In addition, because of global warming, climate change is causing heat waves, droughts, and other abiotic stressors, raising the salt content of the soil in several agricultural areas worldwide [7,8,9,10,11].

The different stressor conditions have led to different environmental and biological limitations that may negatively impact plant growth, yield, and quality [12,13,14,15]. These stresses impact a variety of horticulture crops, causing osmotic stress, water stress, oxidative stress, and ionic strength imbalances that impact several plant physiological, biochemical, and metabolic processes [16]. Different adverse effects of climate change, such as chilling and high temperatures, are experienced by horticulture crops [17]. These effects include oxidative stress, cell membrane breakdown, protein denaturation, and nucleotide damage [18].

Depending on the kind of crops, the stressor, and the duration of exposure, abiotic stress causes several morphological, metabolic, and gene expression alterations in plants [19,20,21,22]. In response to abiotic stress, plant cells produce more free radicals. Roughly 2% of the oxygen taken in is transformed into reactive oxygen species (ROS), which are reactive byproducts of aerobic metabolism that compromise plant metabolism and negatively impact plant production [22,23]. Excessive ROS levels from abiotic stress may harm plant proteins, membranes, and other structural components, inevitably impeding vital physiological functions. Irrespective of stress, some plants have been shown to exhibit membrane peroxidation and damage to their photosynthetic systems. Therefore, scavenging ROS by activating antioxidant molecules is the primary target of plant defense systems [10,22,24,25,26].

One technological advancement crucial in addressing global issues like the depletion of natural resources and the acceleration of climate change is nanotechnology. Enhancing fertilizer use efficiency, reducing plant production losses, and increasing water usage efficiency are critical. Nanotechnology has the potential to improve water and nutrient uptake, antioxidant activity, and photosynthesis, thereby increasing the resilience of horticultural crops to environmental challenges [27,28,29].

Furthermore, it could also control genes that respond to stress and initiate signaling pathways that help plants adapt to stress [10,30]. There has been a rise in the utilization of goods containing nanomaterials, which might mean higher yield quality and productivity. It has been demonstrated that using nanoparticles (NPs) increases plant tolerance to abiotic stress conditions significantly faster than using strategies like genetic improvement [31,32]. NPs offer a better and more comprehensive approach to reducing stress in horticultural crops because of their rapid results and low cost. They also help protect the environment when used carefully, unlike other methods such as genetic improvement, which is time-consuming, or genetic engineering, which is expensive and requires expensive equipment.

Recently, using nanotechnology in agriculture as a substitute for the traditional application of bulk chemical pesticides and fertilizers [33] has the potential to revolutionize sustainable agricultural practices [34]. NPs might provide numerous benefits over conventional agricultural practices, including a large surface area, highly efficient mass transfer abilities, and controlled and targeted delivery of lower nutrient or pesticide amounts, which could improve crop productivity [35]. Nanofertilizers can deliver nutrients more efficiently. Improved uptake by plants due to nanoscale size and surface properties, saving resources and labor. Including a large surface area, highly efficient mass transfer abilities, and controlled and targeted delivery of lower nutrient or pesticide amounts. NPs can be engineered to deliver pesticides in a targeted and controlled-release manner. This means lower quantities of chemicals are needed. Some NPs even help plants tolerate drought stress by modulating physiological responses. Under stress conditions, nanotechnology can improve the efficiency and performance of physio-biochemical and molecular systems in plants [36,37]. Numerous hormone pathways may be triggered or downregulated due to NPs-plant interactions, which can affect plant metabolism and stress responses and control plant growth, development, and stress tolerance [38]. By improving plant tolerance to stress, NPs can enhance crop yields in sustainable agriculture, addressing current and upcoming production challenges [10,17,39]. This can be performed by suppressing plant infections, increasing chlorophyll concentration and photosynthesis’s effectiveness, and activating certain enzymes [21].

This article stresses the necessity for responsible and sustainable usage while examining NPs possible adverse effects and environmental ramifications. This study explores NPs’ role and potential in stress management in horticultural plant species. Furthermore, we look at the influences of NPs on scavenging ROS by activating anti-oxidative enzymes, and how NPs affect fruit quality and production.

## 2. Different Approaches for Nanoparticle Preparation

NPs are molecules engineered typically range in diameter and dimensions from 1 to 100 nanometers (nm). NPs can be classified into the following categories: metallic NPs are zero-dimension nanosized metals (e.g., Zn, B, Cu, Ag, and Se), polymeric NPs are prepared from biocompatible colloidal polymers (e.g., nanospheres or nano-capsules), and non-metallic NPs are prepared from environmentally friendly materials (e.g., CNTs, fullerenes, and graphene) [40,41,42].

**Figure 1:**
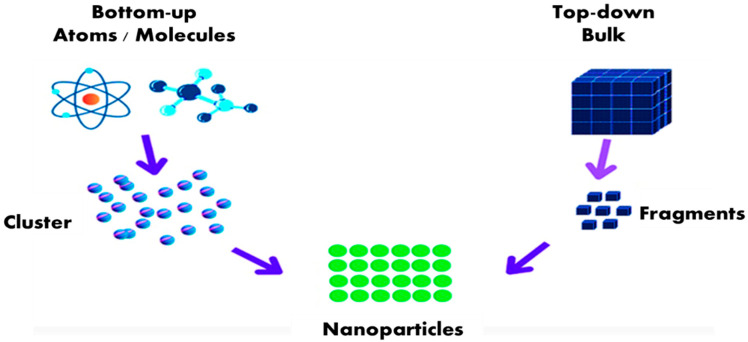
Different ways of preparing nanostructures.

Owing to their enormous surface area and nanoscale size, NPs have particular physicochemical characteristics. Although many different approaches to prepare nanostructures may be broadly divided into top-down and bottom-up methods (Figure 1), metal NPs can generally be synthesized through various methods; chemical ones are the most popular and successful. The materials produced have the benefit of being able to be stored for long periods without exhibiting a discernible loss of stability [43]. It may also be made physically as a metallic nanoparticle by employing ultrasound, microwaves, and irradiation—all of which need substantial energy [44]. Using various biological systems as potential ecological substitutes for physical and chemical methods is another method of synthesis known as biological methods. These methods involve using microorganisms, natural plant extracts, bacterial extracts, enzymes, and plants or plant extracts that can transform metal ions into metal NPs [45,46,47].

In particular, let us briefly discuss some chemical methods used to synthesize metal NPs. For example, we can synthesize magnetite (Fe_3_O_4_) or maghemite (-Fe_2_O_3_) using hydrothermal methods that reduce ferric chloride with ascorbic acid. The resulting product is a black powder with a spherical shape of about 20 nm and a particle size range of 50 ± 5 nm to [48,49].

Ahamed et al. [50] created CuO NPs via the co-precipitation approach, employing NaOH as a reducing agent and copper (II) acetate as a precursor salt. The black precipitate result of the CuO NPs was around 23 nm in size. Additionally, Ganeshan et al. [51] utilized 0.2 M manganese (II) sulfate diluted in distilled water and added NaOH solution until the pH became alkaline, resulting in particles 40–50 nm in size and spherical, crystalline.

Similarly, zinc nitrate hexahydrate reduction by urea at alkaline pH was reported by Abou El-Nasr et al. [52], yielding diameters of spherical NPs at 97.31 nm. Kshirsagar et al. [53] employed the sol–gel technique to dissolve copper (II) chloride in an aqueous solution (0.2 M). They added 1 mL of glacial acetic acid to the solution while stirring continuously and heating it to boil. Next, they added 8 M NaOH to the mixture until the pH reached 7, at which point the size of the copper nanoparticle was 20.4 nm.

It is crucial to understand the distribution, stability, and structure of NPs during the characterization process. Understanding and researching the absorption and transport of NPs in plants is made feasible by tools such as Zeta sizers, X-ray diffraction (XRD), transmission electron microscope (TEM), scanning electron microscope (SEM), and dynamic light scattering (DLS) [54]. Furthermore, absorption efficiency is influenced by several variables, including the kind of plant, its growth stages, the features of the nanomaterial (such as its size, shape, and stability), concentration, and application techniques [17,40,55].

## 3. Nanomaterials’ Uptake in Plants

Plant absorption, or the transport of materials into plants, is an essential process for the growth and development of plants. Plant transport involves the transfer of gases, water, nutrients, and exogenous substances as different NPs [56]. In order to develop the best possible NPs for agricultural usage, it is essential to comprehend how NPs are absorbed and transported by plants [57,58]. Simultaneously, knowing NPs mechanism behind their’ action and their accumulation in plants might assist in elucidating their biological safety and offer recommendations for their safe application [40].

**Figure 2:**
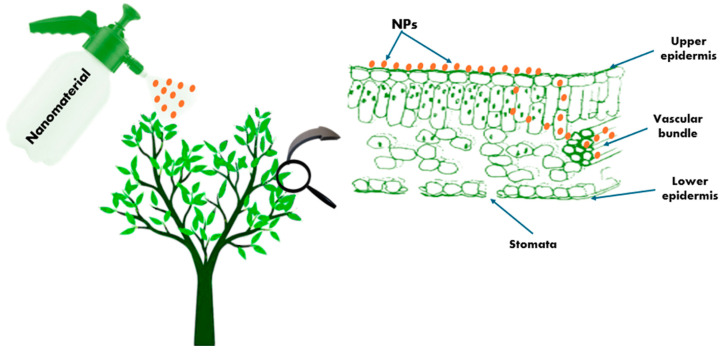
Plant’s absorption and translocation of NPs through foliar spraying.

By administering direct treatment to seeds that have been absorbed during the germination stage, NPs can infiltrate plant tissues. It is immediately absorbed in the subsequent phases through various root tissues, passing through root barriers such as the cortex, endodermis, and epidermis before reaching the xylem and being carried to the vegetative system [54]. Additionally, NPs can boost plant canopy penetration by foliar spray, which avoids the epidermis even if polar compounds are resisted by cuticle, or through stomatal pores [59,60], and diverse plant components [61]. They can enter the vascular tissue via the apoplastic or symplastic pathway. The researchers suggested that the stomatal pathway might be a method by which NPs are absorbed (Figure 2).

Indeed, the particle size and zeta potential of NPs are closely related to their efficiency in penetrating cell membranes. The effect of NPs increases with smaller particle sizes and higher zeta potentials. The size and zeta potential of the resulting particles vary depending on the method of preparing the NPs and the chemicals used.

Materials greater than 100 µm^3^ can only pass through leaves with open stomata when applied topically (foliar) [62]. However, several studies have demonstrated that water-suspended NPs may naturally penetrate via stomata holes with a diameter of less than 100 nm [60] or a pore size of less than 1.1 µm [63]. NPs of 40–50 nm size may readily pass through the cell wall [64]. Osmotic pressure and capillary action aid in the entry of NPs into the cell, and NPs contact with the outermost layer modify membrane proteins as transporters and receptors [17]. Numerous variables, including the NPs’ size, shape, surface characteristics, and the pH and presence of other ions or chemicals in the solution, might affect the NPs’ transportation [56].

## 4. Innovative Solutions in Managing Stress in Horticulture

**Figure 3:**
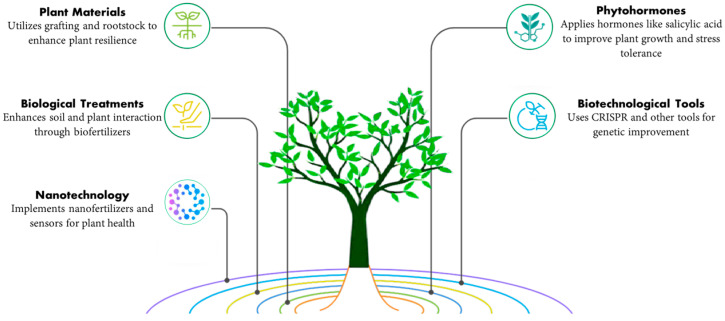
Strategies for stress management in horticulture.

Several effective strategies are followed to manage stress in horticulture crops (Figure 3) as follows:

− Plant materials: Plants have adopted multiple strategies to survive under different stressors used for plant improvement, like vigorous growth, osmotic adjustment, higher water use efficiency (WUE), etc. Rootstocks and improved varieties play an important role in adaptation to stress with different tolerance levels. Grafting is used as an additional tool to alleviate environmental stresses, and this technique is applied to many high-yielding fruits and vegetables such as cucurbits and eggplant to enhance tolerance to saline soil, water shortage, heavy metals, etc. [65,66,67].
− Exogenously applied phytohormones: They are chemical compounds that play an important role in plant extrinsic and intrinsic factors, essential in regulating many signal transduction pathways under stress conditions [68,69] and increasingly accepted by horticultural plants to tolerate stress diversity. Their action is regulated in their metabolism as they are extracted in small amounts from chemical systems [70]. For example, salicylic acid (SA) is one of the hormones that positively influence many developmental processes such as seed germination, seedling growth, photosynthesis, cell reproduction, changes in stomatal aperture, respiration, antioxidant defense system, delaying plant senescence, etc. [71]. SA exposure alleviated chilling injury on the seed germination of muskmelon plants [72] and enhanced the total soluble sugars and cold-response gene expression in peach fruit [73].
− Utilize of biological treatments: Biofertilizers are one of the strategies for agricultural sustainability, especially under stress conditions, as they do not cause any harm or manipulation to the soil microflora, but rather enhance the association between soil, arbuscular mycorrhizal fungi (AMF), and plant growth-promoting rhizobacteria (PGPR) and are effective in developing tolerance to various abiotic stresses or improving nutrient cycling in the soil, thereby enhancing plant productivity [74].
− Use of biotechnological tools: Advances in various biotechnological tools and the development of transgenic lines have been made possible by the introduction of modern molecular or biotechnological tools such as miRNA identification and signaling pathway analysis [75], CRISPR/Cas-mediated genome editing [76], quantitative trait loci (QTL) mapping, and genomic selection (GS), which have made it possible to identify and position the desired gene in the new genome with greater precision, which is involved in different metabolic activities, signaling pathways, and the expression of different genes. This technology helps understand the molecular and physiological mechanisms, stress responses, and improve plant productivity [77].
− Utilization of nanotechnology: It is one of the most promising technologies in mitigating the effects of climate change and enhancing stress management techniques. The application of nanofertilizers via various techniques (e.g., soil irrigation, foliar spray, and seed coating), nanosensors to track the health status of plants in real time, and genetic engineering of plants to boost defense-related phytohormones and photosynthetic efficiency are examples of nano-enabled technologies that have been developed to support plant growth. Several studies have reported using NPs as nanofertilizers to improve plant production under stress conditions [78,79].

Natural resources generally deteriorate, and agricultural production is reduced due to various abiotic pressures caused by climate change, global warming, human activity, and other unavoidable causes [80]. Innovative methods such as using NPs and other technologies are being explored to lessen the adverse effects of these stresses on horticultural plants, as shown in Figure 3. Therefore, understanding these abiotic stresses is essential for creating efficient management strategies and enhancing plant resilience.

## 5. Role of Nanoparticles on Abiotic Stresses

Environmental stressors mitigate the growth, development, and production of plants. Several studies show that plants subjected to various stressors produce ROS, which leads to oxidative damage [81,82,83]. Drought causes wilting, decreased leaf area, decreased photosynthetic activity, and eventually poor development or plant mortality, among other physiological changes in plants. Additionally, it might cause oxidative stress, which damages chloroplasts and mitochondria, causing cellular degeneration. Salinity can result in ionic toxicity, impair cellular processes, and osmotic stress, which restricts plants’ ability to absorb water, with reduced growth rates, necrosis, and chlorosis of the leaves. High temperatures can lead to heat stress, causing protein denaturation, increased respiration rates, and loss of photosynthetic efficiency. Conversely, low temperatures can cause frost damage, impair cellular functions, and reduce metabolic activity. Heavy metals can disrupt plant metabolism by generating ROS, leading to oxidative stress, and impairing enzymatic activities essential for growth and development. ROS are highly reactive oxygen-based molecular intermediates whose metabolism is altered by these unfavorable environments [84]. Antioxidant defense mechanisms guard against oxidative stress, but too much can be detrimental. High concentrations of ROS can damage macromolecules like proteins, nucleic acids, and membrane lipids. This imbalance can lead to nitro-oxidative stress and plant cell death [85,86,87].

NPs are an effective rolling tool for enhancing plant resilience against abiotic stresses. They facilitate various physiological and biochemical responses that contribute to stress alleviation through three primary mechanisms. (I) Improving nutrient uptake and water retention: NPs increase nutrient availability and absorption by enhancing root interaction and improving soil structure, which boosts water retention. For instance, silicon dioxide (SiO_2_) NPs improved the growth of banana plants under drought stress by elevating chlorophyll content and shoot growth [88], while titanium dioxide (TiO_2_) NPs enhanced nutrient uptake and drought tolerance in grape plants [89]. (II) Enhancing antioxidant activity in plants, NPs can induce the expression of antioxidant enzymes, enabling plants to scavenge ROS and mitigate oxidative damage [90]. Research showed that selenium NPs (Se NPs) increased antioxidant enzymes and phenolic compounds in drought-stressed pomegranate plants [91], while iron nanoparticles (Fe NPs) similarly enhanced antioxidant enzyme activity in grape plants [92]. (III) As carriers for stress-mitigating compounds, NPs effectively deliver growth regulators and nutrients, improving their bioavailability and effectiveness [93].

Nanotechnology has great promises for mitigating abiotic pressures and securing the future of agriculture worldwide. It has been shown that NPs can assist plants in withstanding various challenges by improving plant development, growth, and productivity. It is examined here in the ensuing major areas:

### 5.1. Water Stress

Water stressors affected fruit and vegetable crops, in addition to cutting flowers, reduced plant life, productivity, and quality by altering the levels and functions of photosynthetic pigments, osmolyte content, and enzyme activity [94,95,96]. Nanotechnology has recently been incorporated into agricultural systems. Several NP types have been developed for decreased economic and nutritional failures during drought stress, improved yield, and balanced crop production [39].

When applied exogenously to micro-propagated banana cv. “Grand Nain” under drought stress conditions, SiO_2_ NPs at a rate of 50 mg L^−1^ have been shown to increase shoot growth and chlorophyll content, protect cellular membranes by lowering malondialdehyde (MDA) levels, and lessen oxidative damage to plants [88]. Furthermore, when applied to pomegranate plants under drought stress, selenium nanoparticles (Se-NPs) at a dosage of 20 mg L^−1^ and a size of 10 nm increase levels of photosynthetic pigment in leaves, phenolic content, and antioxidant enzymes while decreasing stress-induced lipid peroxidation and H_2_O_2_ content. In contrast to other treatments, severe drought stress also increased the amount of phytohormone accumulated as abscisic acid [31].

Research on vegetables has demonstrated that when ZnO-NPs were applied topically to eggplant (*Solanum melongena* L.) for thirty to forty-five days following transplanting, the plants increased their relative water content (RWC), membrane stability index, and photosynthetic effectiveness [20]. It has been highlighted that exogenous TiO_2_ NPs treatment at 100 mg L^−1^ on tomato plants under drought stress increased stress tolerance, leading to an increase in photosynthesis-related and intrinsic proteins in the plasma membrane, an increase in RWC, and a decrease in MDA, proline, and lipid peroxidation [39,88,94].

When treated with a solution containing 10 mg L^−1^ of silver NPs, some ornamental plants exposed to drought stress, such as cut chrysanthemum flowers, showed an increase in vase life of 3.21 days above the control treatment [97] and prevented vascular occlusion. Additionally, Moringa seedlings treated with 0.1% and 0.05% ZnO-NPs by foliar spray demonstrated a substantial reduction in chlorophyll degradation and increased antioxidant activities and phenolic contents [98].

Table 1 provides an overview of the nanomaterials that improve horticultural crops’ resistance to drought stress by causing biochemical and physiological changes and altering gene expression.

### 5.2. Temperature Stress

Low or high temperatures throughout the growing season can cause a variety of physiological abnormalities in the plant cells, which can negatively impact the quality of the fruit and reduce the rate of photosynthesis and damage to the cell membranes [114].

Severe alterations might harm the intermolecular bonds needed for healthy growth in the summer heat, impeding plant growth and fruit set [115]. Heat stress often shortens the life cycle of plants and lowers output by decreasing the efficacy of photosynthetic activities [116]. Heat stress may become a significant problem limiting productivity in tropical and subtropical regions. When plants suffer heat stress, they produce heat shock proteins and molecular chaperones. A heat shock protein contributes to heat stress resistance and helps other proteins remain stable under stressful conditions. Moreover, multiwall carbon nanotubes promote heat shock protein production and upregulate the gene expression of heat shock proteins [117,118].

**Figure 4:**
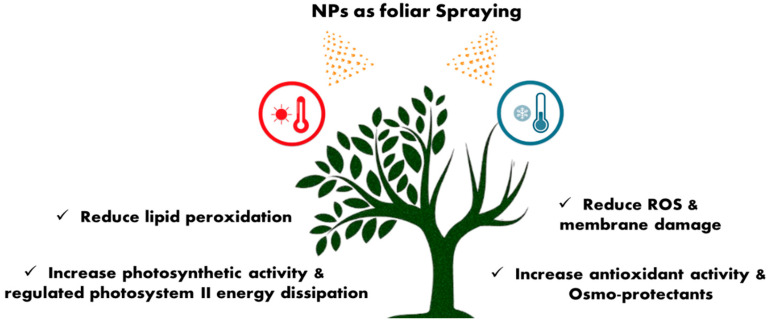
Alleviation of heat stress in plants by nanoparticles.

Excess energy can be released through plants’ stomatal conductance and increasing transpiration rate, which is positively impacted by NPs when under heat stress [119]. Spraying of titanium dioxide nanoparticles (TiO_2_ NPs) was found to be effective in enhancing the physiological and metabolic activities of tomato plants under heat stress by using a low concentration of 50–100 mg L^−1^ [119]. CH-NPs administered topically at lower concentrations could successfully boost banana tolerance to cold stress by reducing oxidative stress through activating the antioxidant [120] as shown in Figure 4.

### 5.3. Salinity and/or Alkalinity Stress

Salinity significantly inhibits plant development and productivity and changes the activities of enzymes [121,122,123,124]. It also severely affects cellular structure by causing Na^+^ and chloride Cl^−^ ions to accumulate in the cytosol [125]. Salt accumulation in the mesophyll cells causes carbon dioxide to be absorbed and the concentration of CO_2_ inside the leaf to rise, which lowers stomatal conductance [126]. By improving antioxidant activities, osmolyte synthesis, and stress-related gene expression, an exogenous application of NPs can mitigate the detrimental effects of salt stress. It can also improve growth-related traits and productivity [113,127].

Furthermore, when subjected to salt stress, the plants adjust and survive by controlling signal transduction pathways and turning on stress-responsive genes [128]. It causes an imbalance in the amounts of nutrients, which causes plants to have severe nutritional shortages [129]. Cu NPs have been demonstrated to support tomato plant development and maintain a balanced Na^+^/K^+^ ratio in the face of salt stress [130].

Based on the current body of research, certain NPs may improve plant development and growth when exposed to saline stress. Applying selenium on lemon verbena “Lippia citriodora Kunth” showed improved salinity tolerance by lowering leaf electrolyte leakage, malondialdehyde, and H_2_O_2_ accumulation, enhancing secondary metabolites’ biosynthesis [131]. Graphene oxide nanoparticles (GO-NPs) can somewhat mitigate the effects of salinity stress. Another study [126] suggested that exposure concentrations of 5 and 10 mg/L of GO-NPs could moderately alleviate the harmful effects of salinity or alkalinity stress in strawberries. GO-NPs improve the morphology and physiology of growth and development in stressed plants by affecting the photosynthetic system and the electron transport chain. Table 2 provides an overview of the nanomaterials that improve horticultural crops’ resistance to salinity stress by causing biochemical and physiological changes.

### 5.4. Heavy Metal Stress

One of the harmful elements of reducing plant production in the modern era is heavy metal stress. Human activities, including industrialization and urbanization, are the global source of heavy metal contamination [138]. The use of chemical fertilizers and pesticides, which are agricultural instruments that negatively impact plants with heavy metals like cadmium (Cd), lead (Pb), cobalt (Co), nickel (Ni), and silver (Ag) [139], has also increased the stress caused by heavy metals in plants. By causing physiological and morphological anomalies as well as metabolic pathway malfunction, heavy metals directly disrupt plant growth performance. This affects the amount and quality of plant production, particularly in vegetable crops and medicinal plants [140].

A few research works have examined how well NPs work to lower heavy metal stress. By absorbing and changing, newly introduced NPs can affect heavy metals in soil by reducing their mobility and bioaccumulation. By releasing phosphate ions, hydroxyapatite NPs can impact the reduction of copper and lead to heavy metal bioavailability while maintaining the pH of the soil at [141]. The quantity of Cd metal in soil has decreased due to Fe_3_O_4_ NPs treatment [142]. By triggering the antioxidant system, NPs can lower ROS by [143].

**Figure 5:**
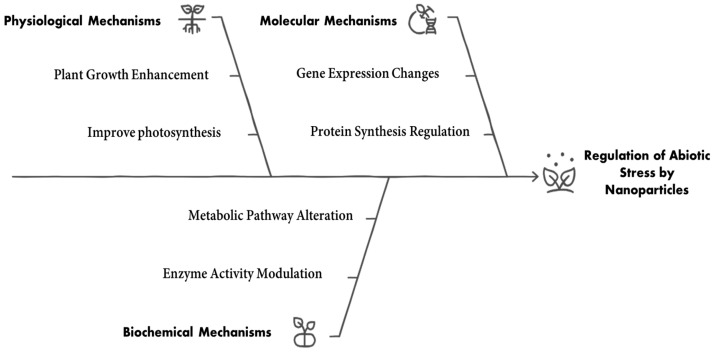
Regulation of abiotic stress by nanoparticles.

For instance, using chitosan NPs as a putrescine carrier, grapevines under Cd stress conditions demonstrated, in unambiguous terms, how NP treatments mitigated the negative effects of stress by increasing antioxidant enzymatic activities, total phenolic compounds, anthocyanins, and lowering Cd content in leaf and root [144]. Similarly, applying 10 mg L^−1^ of Fe_3_O_4_ NPs to coriander plants resulted in a 97.37% and 98.55% reduction in the bioavailability of Cd and Pb, respectively [145]. Furthermore, ref. [146] showed that SiO_2_ NPs increase the activity of the antioxidant enzymes SOD, CAT, and APX, which in turn reduces translocation and partially mitigates arsenic toxicity in tomato plants (Figure 5).

## 6. Role of Nanoparticles on Biotic Stress

Biotic stress refers to the negative impacts on plants caused by living organisms. These stresses can significantly affect plant growth, development, and yield. Key examples of biotic stress include

− Pests: Such as insects and nematodes, that feed on plants and can cause direct damage to plant tissues by chewing leaves, stems, or roots. Common pests include aphids, beetles, and caterpillars. They can also act as vectors for diseases.
− Pathogens: These include fungi, bacteria, viruses, and other microorganisms that can infect plants, leading to diseases. Pathogenic infections can cause various symptoms, including wilting, leaf spots, and root rot. Examples include downy mildew (fungal), bacterial blight, and viral infections like mosaics.
− Weeds: Weeds are unwanted plants that compete with cultivated crops for resources such as light, water, nutrients, and space. They can reduce crop yields and quality by overshadowing crops, harboring pests, and acting as reservoirs for diseases.

It is critical to remember that the NPs’ reactions are dose-dependent and that, when applied improperly, NPs can be phytotoxic to crops, due to the increased concentration of compounds inside the cell and the occurrence of cell toxicity, which results in an increase in free radicals in the cells [147]. They may significantly impact photosynthetic efficiency, apical growth, plant biomass, and seed germination. NPs enhance plant defense by increasing levels of phenolics, activity of antioxidant enzymes in plants, stimulating hormone production like salicylic acid and jasmonic acid, which are key in plant immune responses, enhancing nutrient uptake, improving root architecture, making plants more robust overall, and improving plant resistance against pathogens [148]. NPs demonstrate significant potential in enhancing agricultural practices, particularly pest and disease management. Here is an elaboration on their roles in specific applications:

(a) Delivering pesticides or fungicides in a controlled manner, NPs can encapsulate pesticides or fungicides, allowing for controlled and sustained release [149]. This reduces the need for frequent applications and minimizes the risk of environmental contamination. By improving solubility and stability, NPs can enhance the efficacy of pesticides [150].
(b) Inducing resistance in plants against pathogens, certain NPs can act as elicitors, prompting physiological responses that enhance stress tolerance. Silica or copper-based NPs can stimulate the plant’s innate immune system, activating defense pathways that enhance resistance against pathogens. This may involve increased production of ROS and the accumulation of phenolic compounds, which bolster plant defense mechanisms [151].
(c) Targeting specific pests without harming non-target organisms, NPs can be engineered to be selectively toxic to specific pests while minimizing effects on beneficial and non-target organisms [152]. For instance, targeted delivery using lipid-based NPs can affect pest behavior without adversely impacting pollinators like bees [153].

Through NPs’ ion release, which damages DNA by interacting with bases in DNA, they can directly impact plant homeostasis. Alternatively, they can have indirect impacts by producing ROS, which alters the activity of antioxidant enzymes. By improving plant nutrition, reducing pathogen infections (bacterial, fungal, and viral), and raising crop output and nutrition quality, many NPs can directly combat pathogens or strengthen defenses against illnesses [148]. They primarily suppress the plant’s ROS response and promote plant growth and development by regulating the antioxidant systems, endogenous plant hormones, and the transcriptional regulation of stress-related genes [154,155]. In terms of direct pathogen-attacking activity, distinct NPs employ various antibacterial mechanisms. Agriculture may use a variety of nanomaterials, with metal or metal oxide NPs like Ag NPs and CuO_2_ NPs, as well as lipid or polymer-based NPs, such as micelles and chitosan nanoparticles (CS NPs), being particularly useful [148].

There is a significant distinction between the two primary kinds of NPs (organic and inorganic NPs) in terms of applications that center on biotic stress: (i) Inorganic NPs often have inherent action against the pathogen (Ag NPs, for example, directly exhibit antifungal and antibacterial activity against bacteria, fungi, and nematodes that infect plants) [155]. (ii) The primary use of polymeric nanoparticles (PNPs) is as nanocarriers, which facilitate the effective release of the active drug [154] and improve bioavailability. In recent years, PNPs have been the subject of much research due to their potential use in controlled release and environmental protection of active substances. There is much interest in using PNPs in agriculture because of their excellent stability and capacity to release active chemicals in a specific plant target zone. Moreover, PNPs have minimal toxicity due to their biodegradability and biocompatibility. Furthermore, these NPs can contain many active substances with minimal environmental influence because of their delayed release. The usefulness of PNPs is therefore frequently dependent on their combination with other active compounds, such as antibiotics, herbicides, insecticides, or even micronutrients (Table 3) [155].

### 6.1. A Polymeric Nanoparticle

With lower quantities of the active ingredient, PNPs often possess the ability to transport molecules efficiently in agriculture. Consequently, there is less soil leaching, better thermal and photostability, and higher adhesion and absorption [148]. Given these factors, commonly produced PNPs for agricultural applications are best composed of low-cost, biocompatible, and biodegradable polymers with low toxicity. Because of its cost-effectiveness, biocompatibility, and biodegradability, chitosan is the most often used biopolymer in agricultural applications [159].

ROS generation, elevated antioxidant enzyme activity (catalase, glutathione peroxidase, peroxidase, and superoxide dismutase), and antioxidant biomolecules (flavonoids) all support plant defense against biotic stress [160]. An inventive technique for reducing the severity of leaf rust disease included misting salicylic acid and chitosan NPs before and during plant leaf inoculation with *Puccinia striiformis*, an essential fungal parasite [160].

In addition to chitosan, atrazine, a pesticide that works against the weed species *Bidens pilosa*, was included in nano-capsules made of the natural polymer poly [161]. Encasing the herbicide in PCL NPs reduced the genotoxicity of atrazine and its soil mobility. PCL containing atrazine was useful in suppressing agricultural weeds while lessening the herbicide’s toxicity [161].

It has been possible to produce new and efficient nanocarriers to lessen biotic stress in agriculture by employing various ingenious and intriguing techniques. Sustainable development principles guided the creation of pesticides (such as the model insecticide Azox) utilizing renewable plant oil-based polymers [162]. Promoting the sustainable release of agrochemicals is another use for NPs based on alginate [162].

### 6.2. Metal Nanoparticles

Silver NPs are effective nanopesticides against a variety of phytopathogens, including *Fusarium oxysporum*, *Cladosporium cucumerinum*, *Pyricularia oryzae*, *Alternaria alternata*, and *Sclerotinia sclerotiorum* [163,164,165]. They work by various methods, such as ion release, creating pits and holes in bacterial membranes, and interacting with enzymes’ disulfide or sulfhydryl groups, impairing metabolic processes [160].

Besides their antibacterial properties, silver nanoparticles (Ag NPs) enhanced the germination of seeds and altered the biochemical composition of pearl millet [166]. This included increased phenol, flavonoid, and protein content, and peroxidase and superoxide dismutase activity [167]. Cu NPs are also highly effective nanopesticides that improve plant nutrition and development. Their antibacterial action includes disrupting essential enzymes and removing NPs from the bacterial cell membrane [168]. Important phytopathogens like *Fusarium oxysporum*, *Aspergillus niger*, *Rhizoctonia solani*, *Xanthomonas axonopodis*, *Fusarium destructiva*, *Curvularia lunata*, *Alternaria alternata*, *Clavibacter michiganensis*, *Gibberella fujikuroi*, and *Drechslera sorghicola* are among those against which they exhibit antimicrobial activity [168].

Cu NPs applied topically to foliage enhanced growth and chlorophyll content while reducing Fusarium wilt incidence and severity by 68 and 66.5%, respectively [169]. Another study found that applying Cu NPs topically to plants improved the quality of their fruit by encouraging the build-up of bioactive substances such as flavonoids, lycopene, vitamin C, and total phenols [168]. Apart from these two NPs, TiO_2_ NP, a metal-based nanoparticle, exhibits potential uses in reducing biotic stress in various plants. Producing ROS and lipid peroxidation can induce oxidative stress, damaging pathogen cell integrity and increasing membrane fluidity [160].

Because silica NPs directly affect plant development, they offer enormous agricultural potential [170]. The increased absorption and translocation of silica by silica-based nanomaterials decreases the production and build-up of ROS and lipid peroxidation, enhancing resilience to biotic and abiotic stressors [171]. In addition to serving as nanocarriers for proteins, nucleotides, and other active molecules in agriculture, empty silica NPs can be employed as biostimulants, nanofertilizers, herbicides, and insecticides [172]. By reducing the amount of sodium ions and heavy metals that enter plants, nanosilica reduces salinity and the toxicity of heavy metals.

The accumulation of nanosilica in plant leaves significantly strengthens the plants’ resistance to infections. Not only can nanosilica bio-stimulate plant cells, but it also possesses antibacterial and antifungal characteristics. Furthermore, it is known that nanosilica stimulates the production of genes involved in defense. Nanosilica can induce acquired resistance in plants, a plant immunological response that greatly enhances plant defense against biotic stress [173].

According to a study by [173], silicon-based NPs have been created and employed as pesticide agents and nanocarriers in plants to guard against diseases. Lately, spherical silicon nanoparticles (NPs) with a size of 45 nm and a negative zeta potential of −26 mV, produced by Fusarium oxysporum SM5, showed nematicide effects due to the presence of meloxydone [170]. When sprayed in a concentration of 100 mg/L, the silica NPs significantly reduced the damage caused by *Alternaria solani* and demonstrated in vitro antifungal activity against the fungus. Furthermore, a notable increase in the content of antioxidant metabolites and enzymes was noted. The authors found a notable rise in antioxidant metabolism, a notable drop in infection damage indices, and a more noteworthy production of chemicals linked to defense against biotic stress [170,173].

However, research showed that silicon NPs prevented egg hatching, and after 72 h of exposure to NPs at 100 and 200 ppm, the death rate of second-stage juvenile root-knot nematodes varied from 87% to 98.5%. Interestingly, commercial nematicides at half-recommended doses combined with silicon NPs with 100 ppm further reduced egg hatching and second-stage juvenile root-knot nematode mortality. Based on these findings, it may be possible to combine silicon NPs with conventional nematodes to control pathogens in agricultural production [173]. Additionally, the therapy enhanced the nematode-invaded plants’ growth and ability to absorb vital nutrients.

Selenium nanoparticles (Se NPs), in addition to nanosilica, are recognized as an environmentally and ecologically benign method of boosting plant yield by reducing abiotic stressors since Se triggers plant defense systems [174]. Because biosynthesized Se NPs may produce ROS, which destroy pathogen cell walls, degrade the integrity of pathogen cell membranes, and limit ATP synthetase activity, they have antibacterial effects against phytopathogens such as fungi and bacteria [175]. By breaking down the cell wall and changing the cycle of food metabolism, protein synthesis, modification, and deoxyribonucleic acid replication, Se NPs can also directly prevent the growth of pathogens, ultimately leading to the death of the microbes [174,175].

### 6.3. Metal Oxide-Based Nanoparticles

Another metallic NP to be aware of is oxide-based NPs. In this regard, *Botrytis cinerea*, which causes gray mold, was 80% stopped from growing by photoactivated ZnO NPs. ZnO NP spraying on strawberries improved plant yield by 28.5%, prevented harvested fruit deterioration during storage by 8 days, and decreased the incidence of *Botrytis cinerea* by 43% [176].

An alternative method involved inoculating the soil with ZnO NPs generated by *Matricaria chamomilla*, which reduced the number of *Ralstonia solanacearum* that causes bacterial wilt and the severity of the illness while promoting better plant development. Because of the likely release of Zn^+2^ ions, the bacterial cells exhibited morphological deformation, including breakage of the cell wall and membrane and leaking of cell contents [177,178]. Free radicals can accumulate due to a change in the redox balance of plant cells. These radicals can alter cell signaling pathways and oxidative damage macromolecules [177]. In addition to metal-based and polymeric NPs, several notable NPs have been successfully studied in agricultural applications to alleviate plant stress.

SiO_2_ NPs, or nanosilica, have become important in plant defense against abiotic stressors. Nanosilica’s excellent surface-to-volume ratio and biocompatibility contribute to its widespread biological use [179]. Because of its nanoscale size, which enables quick absorption by the apoplastic route and translocation in plant tissues and as a nanocarrier for active chemicals, nanosilica exhibits better plant effects than bulk silica [179]. It is commonly known that nanosilica has a positive effect on helping plants develop stress tolerance. Although the beneficial effects of nano silicon have been well-documented, ref. [180] noted that the effect varies depending on many factors, including the type of application (usually foliar or to the substrate), the concentration and bioavailability of nano silicon, and the plant species used as a biological model.

The rust-causing bacterium *Ustilago tritici* was shown to be susceptible to antimicrobial action by biogenic TiO_2_ NPs made from *Chenopodium quinoa* leaf extracts, which suppressed up to 75% of mycelial growth [169]. Additionally, Satti and associates demonstrated that TiO_2_ NPs made from Moringa oleifera leaf aqueous extract exhibit antimicrobial activity against *Bipolaris sorokiniana*. This pathogen causes spot blotch disease, stabilizing the plant’s chlorophyll content, soluble sugar, protein, proline, flavonoid, and phenolic contents to induce disease tolerance in wheat plants [181].

### 6.4. Carbon Nanomaterials

Carbon-based NPs are a significant type of nanomaterial that can help plants under biotic stress. Atoms of carbon (C) may arrange themselves into various configurations. A stack of two-dimensional single sheets called graphite is created when C atoms are arranged as a honeycomb lattice. The atomic structure of graphene is composed of a single graphite layer [182]. Carbon nanotubes (CNTs) are another carbon-based structure that may be considered a single graphene sheet rolled down an axis parallel to the graphene crystallographic orientations [183].

Nanomaterials based on carbon have also shown practical antiviral, antifungal, and antibacterial qualities. Reference [184] reported that a carbon-based nanomaterial exhibited significant antibacterial activity while preventing the development of biofilms. Plants can employ CNTs as antibacterial agents. For example, CNTs lessened *Alternaria solani* harmful impact on plants. Through both direct antibacterial action and induction of the plant antioxidant defense system, this illness results in plant losses [185]. Giving plants CNTs increased their flavonoid concentration, ascorbic acid content, and glutathione peroxidase activity. Based on the production of ROS following nanomaterial absorption by plant tissue, CNTs have antibacterial action that lessens the severity of *A. solani*.

## 7. Nanoparticle Interaction with Plants

The role of NPs in mitigating abiotic or biotic stress of plants is related to the composition and structure of cell walls. Some elements are involved in the structure and functioning of the cell wall and membrane. Therefore, it affects the metabolite transport reactions. It can influence the signaling pathway that regulates cell expansion, which is associated with stress resistance [186,187]. The interaction of NPs with plants is complex and can have both beneficial and detrimental effects. These interactions are influenced by the NPs’ properties and the plants’ physiological status. The interactions occur at a molecular level through various mechanisms that can significantly affect their physiology and biochemistry as follows:

(a) Uptake Mechanisms: NPs can be absorbed by either endocytosis or passive diffusion by plant roots. For instance, Ref. [173] found that gold nanoparticles (Au NPs) could penetrate root cells through various mechanisms, including apoplastic and simplistic pathways. Negatively charged NPs were observed to gradually accumulate in protoplasts and roots, eventually becoming noticeable in the root xylem, while positively charged particles accumulated on root surfaces and were not absorbed by either [188]. (b) Cellular Interactions: NPs interact with the composition of the cell wall, which consists of cellulose, hemicellulose, and pectin. Their small size allows for easy penetration of the cell wall. Several literature data indicate that the cell wall pore size ranges between 2 and 20 nanometers. It is noted that NPs smaller than 20 nanometers easily pass through the cell wall [189]. The cell wall’s composition can influence nanoparticle attachment and penetration [190]. (c) Biochemical effects: Certain NPs can induce stress by generating ROS in plant cells, leading to alterations in growth and metabolism, and confer stress tolerance by modulating oxidative stress responses [191]. (d) Gene expression: NPs can affect gene expression related to stress response pathways, growth regulation, and metabolism [157,192]. NPs strengthen antioxidant activities and shield plants from oxidative stress by causing the accumulation of antioxidant genes, osmolytes, nutrients, and amino acids [193]. (e) Toxicological impacts: In some cases, NPs can be toxic to plants, leading to cell damage or death [194], it has been discovered that NPs immediately interact to the cell membrane and organelles, causing harmful ions to dissolve and be released, ROS to be produced, and oxidative stress to follow [193]. (f) Transport and Distribution: NPs can be translocated to various plant parts (leaves, stems), affecting overall plant health and physiological processes [56].

Also, NPs play a multifaceted role in modulating various physiological and biochemical processes in plants. Such as growth promotion, improving nutrient uptake and bioavailability, facilitating better absorption by plants [195], bolstering plants’ resistance to abiotic stresses [196], enhancing chlorophyll content and photosynthetic efficiency [197], regulating phytohormone signaling [198], and modulating responses to environmental stimuli [199].

On the contrary, excessive concentrations of NPs can cause phytotoxicity, which affects seed germination and biomass production, interferes with the photosynthetic system, produces oxidative stress, damages cell membrane integrity, changes gene expression, damages DNA, and results in epigenetic alterations in plants [193].

## 8. Biosafety of Nanomaterials

**Figure 6:**
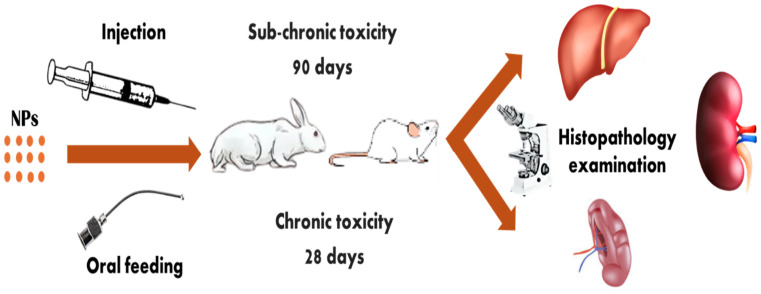
Chronic or sub-chronic toxicity of nanoparticles on experimental animals.

The expanding use of nanotechnology in agriculture prompts enquiries and concerns about its possible impacts on human and environmental health [200,201]. Therefore, it is imperative to study the residual effect of nanomaterials in fruits on experimental animals as a mammalian model to determine the degree of potential harm that these items may cause. Follow ethical and scientific considerations during animal studies to avoid undue suffering of animals [202]. Nanoparticle toxicity is assessed by long exposure; for acute/sub-chronic toxicity, exposure times vary from 28 to 90 days [203]. Oral feeding or injection are the treatment methods [204], and a histopathological analysis is conducted to ascertain the level of toxicity as shown in Figure 6.

With little or almost no research on this point, we review the most important potential effects of nanomaterials on experimental animals’ growth, development, and histological alteration. At low dosages, NPs have much potential that impacts animal nutrition, growth performance, and overall health [205]. NPs help animals achieve their nutritional needs, increase their production, boost their immune system and microbial profile, and lower their risk of illness, but may increase cellular absorption and translocation inside an animal’s body, which might impact their toxicity [206]. Inflammation or cell death may result from NPs at the cellular level and can cause pathological alterations in the liver, pancreas, kidney, intestine, brain, and adrenal glands [207].

According to [208], selenium NPs have positive effects on liver function and lower blood sugar levels and alanine aminotransferase (ALT), aspartate aminotransferase (AST), gamma-glutamyl transferase (GGT), and alkaline phosphatase (ALP) levels. Furthermore, in people with diabetes, selenium NPs may lower blood levels of renal components such as albumin, urea, and creatinine.

According to [209], the effects of prolonged exposure to gold nanoparticles (Au NPs) on the structure and function of the liver, spleen, and kidneys have been investigated to determine the main characteristics of Au NP accumulation in the tissues that may contribute to their toxicity. The function of the liver and spleen was unaffected by the long-term buildup of Au NPs in these organs. However, modifications in these organs’ structure, such as higher macrophage infiltration and higher fibronectin levels, indicated a toxic reaction linked to long-term exposure to Au NPs. It is necessary to clarify whether these alterations are temporary and will go away with time, or if they are the first of a progressive inflammatory disease.

## 9. Real-World Applications of Nanoparticles in Horticultural Plants

NPs have found numerous applications in horticultural plants, primarily aimed at enhancing growth, improving pest and disease resistance, aiding nutrient delivery, and promoting sustainable agricultural practices. Key practical uses of NPs in horticulture crops include the following:

(a) Improvement of Nutrient Uptake: NPs can formulate slow or controlled-release fertilizers. Nanostructured fertilizers enhance nutrient absorption and reduce loss due to leaching.
(b) Disease and Pest Management: NPs can enhance the efficacy of pesticides by improving their delivery to the target site while reducing the required dosage. For instance, chitosan NPs encapsulating pesticides have shown effective control of pests in crops like cucumbers [210].
(c) Stress Mitigation: NPs can mitigate abiotic stress effects in horticultural crops, such as drought and salinity. For instance, silica NPs enhance drought tolerance in plants like cucumber, improving overall plant health and yield [211].
(d) Growth Enhancement: NPs can be used in seed priming to improve germination rates and seedling vigor. For example, zinc oxide NPs have enhanced germination rate and early growth in crops [212].
(e) Improved Photosynthesis: Certain NPs, such as titanium dioxide (TiO_2_), can enhance photosynthetic efficiency and chlorophyll content in horticultural crops. For example, TiO_2_ NPs have been shown to improve growth and increase net photosynthetic rate in *Mentha piperita* L. [213].
(f) Disease Resistance: NPs can induce systemic plant resistance against diseases. For example, selenium NPs have been found to induce defense responses against fungal pathogens in crops like strawberries (*Fragaria* × *ananassa*) [154].

The application of NPs in horticultural crops shows great promise for enhancing agricultural practices by improving nutrient uptake, pest and disease management, stress tolerance, and overall crop yield. As research advances, these applications can lead to more efficient and sustainable horticultural systems.

## 10. Advancements in Nanotechnology-Enabled Stress Management Techniques

Advancements in nanotechnology have introduced innovative stress management techniques that enhance the resilience and productivity of crops under various stresses. Here are some notable advancements in nanotechnology-enabled stress management techniques:

(a) High dispersibility, heavy metal-free nanomaterials

Despite the potential benefits of nano-enabled plant stress tolerance, heavy metal-containing NPs, such as quantum dots Ag and with Cd^2+^ may cause environmental harm and biosafety issues [214]; the potential for NPs to aggregate on plants, which might hinder their ability to respond to stress, is an additional problem [215]. Aggregated NPs have less surface area and cellular absorption than dispersed NPs, frequently leading to toxicity. Therefore, the development of nano-enabled agriculture requires the usage of suitable nanomaterials [216]. The adoption of NPs for agricultural practices and consistent biological activity would be supported by improving the dispersion quality of the materials to prevent agglomeration after application [217].

(b) Nanosensors for detecting stress

Stress sensing and signaling are two of the many complex defense systems plants have developed to withstand stress. Plant signaling molecules include H_2_O_2_, Ca^2+^, gas molecules (nitric oxide, carbon monoxide, and hydrogen sulfide), plant hormones (ethylene, methyl salicylate, jasmonic acid, and abscisic acid), sugars (glucose and sucrose), and volatile organic compounds (isoprenes) [216,218]. In plants under salt stress, signal transduction from root to shoot involves Ca^2+^ signaling [219,220]. The ratiometric quantum dot sensor for glucose [214], hemin-complexed DNA aptamer-coated single-walled carbon nanotubes for H_2_O_2_ [221], are examples of plant nanobiotechnology’s effectiveness in monitoring signaling molecules in different plant species.

(c) Nano-encapsulated

Developing nano-encapsulated fertilizers and nutrients allows for targeted delivery, mitigating stress effects due to nutrient deficiencies. Nano-encapsulation is an innovative and promising nanotechnology that allows the active ingredients to be released from capsules or particles in a controlled and progressive way [222]. Studies have shown that the exogenous application of chitosan NPs encapsulated spermine alleviated the adverse effect of salinity on chili pepper plants and enhanced growth, yield, chlorophyll content, and antioxidant enzymes [223].

## 11. Future Perspectives

Ongoing research in nanotechnology applied to agriculture is rapidly advancing, focusing on various aspects such as enhanced crop protection, precision agriculture, efficient resource usage, sustainability, disease resistance, plant immunity, and soil remediation. Nanotechnology is expected to be a transformative technology that enhances many aspects, with increasing uses of nanomaterials in agricultural applications and consumer products. In addition, it has become a fascinating way to enhance plant development under biotic and abiotic stress situations, especially in light of sustainability. Using NPs can greatly enhance plant development and, in the event of biotic stress, might lessen the detrimental effects of phytopathogens. In agriculture, biotic stress remains a major contributor to yield loss, accounting for 20–40% of losses due to pests and diseases [177]. In addition to carrying compounds with long-lasting antibacterial properties, nanomaterials can be directly harmful to plant pathogens [167].

Despite significant advancements in the application of nanomaterials in agriculture, specific crucial points still require clarification. NMs have been the subject of in-depth experimental or pilot research. To confirm their possible long-term effects on the soil and its microbiome, plants, and animals, further research at the scale of hectares of agricultural fields and the scale of several years of treatments is still required [168]. Further data is also required on the safe and viable commercial application of NMs and the safety of plants grown with their help [162]. One way to emphasize the difficulties in using nanomaterials to counteract biotic stress is the need for more thorough assessment of the absorption, transport, and alteration of nanomaterials in plant tissue. Numerous factors, including the chemical makeup of the material, size distribution, surface charge and chemical surface, form, dose, concentration, application method, length of treatment, interactions between the material and the target plant, and environmental microbiota, all have a significant impact on how nanomaterials affect plants [103]. As anticipated, nanomaterials have beneficial effects at low concentrations while becoming harmful at higher dosages or concentrations. NPs can serve several purposes because of their well-researched capacity to break down hazardous substances found in the environment [112].

Areas needing more investigation to assess how NPs influence the diversity and functioning of microbial soil communities over prolonged periods. Changes in microbial populations can affect nutrient cycling and soil fertility [154]. Also, understand how plants take up NPs over time and their accumulation in edible plant tissues. The long-term implications for food safety and human health remain largely unexplored. In addition, understanding the persistence of NPs in soil and water is crucial for evaluating their long-term environmental risks.

Despite the obvious advantages of nanomaterials, there are still unanswered problems regarding the potential environmental effects of the NPs utilized in daily life [224]. A significant additional obstacle is the public’s acceptance of this technology, as people and/or animals are the ultimate users of plants treated with nanomaterials, and it is necessary to assess the impact of these technologies on human and animal health. NPs may accumulate inside cells and cause intracellular alterations, including gene mutations or organelle damage [225]. Therefore, it is necessary to evaluate nanomaterials’ toxicity and biological activity on experimental animals as a mammalian model. Ecotoxicological research on nanomaterials and establishing a regulatory framework for their use in large-scale agricultural production are crucial steps in this approach. We welcome more research to improve the safe application of nanomaterials on a large scale in the agricultural industry.

## 12. Conclusions

Nano-bioengineering is a significant future research interest that could improve food security and crop production under biotic stress. The main challenge to developing horticultural crops today is climate change. This has resulted in significant losses in horticultural crops, which are an important source of income for those employed in the agricultural sector because they are a fundamental component of food security, as well as in the pharmaceutical and many other industries. In this work, we have reviewed the types of NPs and the different methods of preparation (top-down and bottom-up approaches), emphasizing their potential application in agriculture to improve stress resilience in horticultural plants. Also, the mechanism of action and interaction between NPs and plants at the molecular level facilitates uptake and translocation. They can penetrate plant tissues through root systems or foliar application, enhancing nutrient absorption and providing protective effects against ROS generated during stress. The article explores how NPs can alleviate various biotic and abiotic stresses. They achieve this by enhancing antioxidant defenses, regulating osmotic adjustment, and promoting healthy growth through improved nutrient delivery and signaling pathways. The review underscores the necessity for responsible and sustainable usage of NPs, highlighting potential ecological impacts and the importance of ecotoxicological studies in promoting safe agricultural applications. Ultimately, the review concludes that nanotechnology offers innovative solutions to some of the most pressing challenges facing food production, sustainability, and environmental health. Their unique properties at the nanoscale can enhance crop yields, improve pest and disease resistance, optimize resource use, and contribute to efficient soil and water management, with the need for further research and responsible application.

## Figures and Tables

**Table 1:** Nanomaterials and their impact on horticulture crops under drought stress conditions.

Horticulture Crop Species	Nanomaterials/Addition Method	Effects	References
Mango *Mangifera indica* L.	Selenium (Se), Titanium (Ti), and Silicon (Si) NPs Foliar spraying of Se NPs (5, 10, and 20 mg/L); TiO_2_ NPs (40, 60, and 80 mg/L); SiO_2_ NPs (50, 100, and 150 mg/L).	Compared to the unsprayed plants during the experimental seasons, NPs significantly enhanced the vegetative parameters of mango trees during drought, improving the metrics related to fruit quality and ultimate production.	[99]
Grape *Vitis vinifera* L.	Iron nanoparticles (Fe NPs) Added Fe NPs with different concentrations (0, 5, 10, 20, 30, and 40 µM) dissolved in half-strength Hoagland solution without Fe-EDTA, under simulated drought stress by adding 7% Polyethylene glycol (PEG) to the growth.	The plants’ physiological integrity was saved by increasing the generation of H_2_O_2_ and MDA, which decreased leaf RWC, chlorophyll content, and chlorophyll fluorescence (Fv/Fm). This was notably true at 30 µM Fe NPs, the ideal antioxidant concentration.	[100]
Pomegranate *Punica granatum* L.	Silver nanoparticles (Ag NPs) Sprayed three times at the initial flowering, full bloom, and one month later with 7.5 and 10 mg L^−1^	Enhanced the amount of bioactive chemicals and improved fruit quality, production, and biomass.	[101]
Strawberries *Fragaria* × *ananassa* Duch.	Titanium nanoparticles (TiO_2_ NPs) Application of NPs by foliar spray at 0, 10, 20, and 30 mg/L^−1^	Enhancing the productivity of plants under drought stress by TiO_2_ NPs is a convenient strategy. TiO_2_ NPs led to an increase in flavonoids.	[102]
	Nano-Silicon Dioxide (SiO_2_ NPs) Adding SiO_2_ NPs at (50 and 100 mg L^−1^) in an MS medium containing PEG at different concentrations, plantlets were cultured for 35 days.	Enhanced resistance to water stress through an increase in the weight and length of the roots, the number of leaves, the SPAD index, the CAT, and the SOD activity.	[103]
	Iron nanoparticles (Fe_3_O_4_ NPs) The treatments consisted of Fe_3_O_4_ NPs at 0.08 and 0.8 ppm, salicylic acid (SA) at 0.01 and 0.05 mM; combined effect on branch number and other phenotypical traits, under drought stress at three levels, 5 and 10% simulated by PEG.	In vitro cultivation of strawberry plantlets treated with Fe_4_ NPs and SA improved the quantity and quality of morphological and growth metrics while reducing the adverse effects of drought stress.	[104]
Tomato *Solanum lycopersicum*	Nano-nutrients solution of biochar (NNS) Foliar was applied thrice, with 0%, 1%, 3%, and 5%, after two weeks of drought stress.	Increased plant biomass by the exogenous NNS administration, lessening the effects of drought-induced oxidative stress. It improved membrane stability, decreased the buildup of ROS, decreased lipid peroxidation, elevated levels of secondary metabolites, osmolytes, and antioxidant enzymes.	[105]
	Nano-vermicompost (NV) After germination, seedlings within 20 days were transplanted into pots filled with soil and NV at 10 and 100 mg kg^−1^ of soil. Then it is exposed to drought stress for fifteen days.	Lipid peroxidation, decreased ROS formation, and improved membrane function were the results of NV supplementation. Drought-mediated damage was also prevented by strong modulation of the antioxidant system, osmolytes, and secondary metabolites.	[106]
Potato *Solanum tuberosum* L.	Zinc oxide (ZnO NPs) and Magnetite (Fe_3_O_4_ NPs). Adding NPs at 0.0, 2.5, and 5.0 ppm under drought stress simulation by Sorbitol at 0.0, 1, 2, 3, and 4 Mm on micropropagation, micro tuberization, and some biochemical characters using potato plantlets.	Increased the accumulation of secondary metabolites such as quercetin and kaempferol and their ability to scavenge the radical DPPH (2,2-diphenyl-1-picrylhydrazyl).	[107]
Okra *Abelmoschus esculentus* L.	Iron Nano-Chelate (Fe N-C) Application of nano fertilizer at 3.5, 7, and 10 kg ha^−1^, under drought stress at 100%, 80%, and 60% of soil field capacity.	Plant metabolism increased due to Fe N-C, and nutrients were absorbed into plant tissues more effectively.	[108]
Eggplant *Solanum melongena* L.	Zinc Oxide Nanoparticles (ZnO NPs) ZnO NPs concentrations (0, 50, and 100 ppm), under full irrigation (100% crop evapotranspiration; ETc) and drought stress at 60% ETc.	ZnO NP-treated water-stressed plants displayed enhanced growth traits, increased productivity, and a reduction in the effects of drought stress. These enhancements included better anatomical features of the stem and leaf, more photosynthetic efficiency, and higher RWC and membrane stability index.	[20]
Coriander *Coriandrum Sativum* L.	Silicon nanoparticles (Si NPs) Si NPs and Si-bulk at 1.5 mM were foliar-applied three times at an interval of 15 days.	Total phenolic content (TPC), total flavonoid content (TFC), and optimal essential oil (EO) quality and quantity all increased after Si NPs treatment.	[109]
Pumpkin *Cucurbita pepo* L.	Multi-walled carbon nanotubes (MWCNTs) Using MS medium containing 3% sucrose, and 0.8% agar without or with MWCNTs at different concentrations of 125, 250, 500, and 1000 µg mL^−1^ for germination, under water stress that was simulated by adding PEG at 150 g/L of medium.	MWCNTs protect seedlings from oxidative damage by increasing the activity of antioxidant enzymes at low doses. These enzymes scavenge excess ROS. However, elevated oxidative damage markers, such as MDA and H_2_O_2_, were seen at high concentrations.	[110]
	Nano-potassium (K NPs) K NPs at 0.5, 1.5, and 2.5 ppt were sprayed three days before irrigation.	Using K NPs improved plant development and sped up the leaves’ absorption and transfer of elements, which proved successful. Using K NPs improved plant development and sped up the leaves’ absorption and transfer of elements, which proved successful.	[111]
Russian Sage *Salvia abrotanoides* Kar.	Chitosan nanoparticles (CNPs) CNPs at 0, 30, 60, and 90 ppm, under multiple irrigation regimes, 30%, 50%, and 100% of field capacity.	By enhancing RWC, total chlorophyll, carotenoids, phenol, flavonoids, soluble sugar, proline, and protein, CNPs lessened the impacts of drought stress. Additionally, it increased SOD, PPO, and GPX activity. Furthermore, stomatal density increased, and stomatal aperture size decreased.	[112]
Rose periwinkle *Catharanthus roseus* L.	Chitosan nanoparticles (CNPs) Irrigation regimes of 50% and 100% of field capacity with foliar application of CNPs (0–1%).	By increasing proline accumulation and CAT and APX activity, as well as lowering H_2_O_2_ and MDA accumulation and alkaloid biosynthesis gene expression.	[113]

**Table 2:** Nanomaterials and their effects on horticulture crops under salinity stress conditions.

Horticulture Crop Species	Nanomaterials/Addition Method	Salinity Level	Effects	References
Grape *Vitis vinifera* L.	Chitosan-salicylic acid nanocomposite (CS-SA NCs) Foliar spraying of NCs (0, 0.1, and 0.5 mM).	Three levels (0, 50, and 100 mM NaCl)	The concentration of NCs at 0.5 mM had a better effect and improved the grapes’ physiological and biochemical properties by enhancing total soluble protein, soluble carbohydrate, total antioxidant, and antioxidant enzymes activity.	[132]
Mango *Mangifera indica* L.	Nano-zinc oxide (nZnO) and nano-silicon (nSi) Foliar spray of nZnO (50, 100, and 150 mg/L), nSi (150 and 300 mg/L), and the combination was applied at full bloom and one month after salt stress.	The soil salinity was 3.67 dSm^−1^, and the salinity of the irrigation water used was 0.96 dSm^−1^.	The combined application of 100 mg/L nZnO and 150 mg/L nSi showed improved nutrient uptake, carbon assimilation, plant growth, productivity, and fruit quality.	[133]
Strawberry *Fragaria* × *ananassa* Duch.	Zinc oxide nanoparticles (ZnO-NPs) Application of ZnO-NPs at (0, 15, and 30 mg L^−l^).	Three levels of NaCl-induced salt stress (0, 35, and 70 mM)	The lower concentration at 15 mg l^−1^ was found to alleviate the harmful effects by enhancing the growth traits, decreasing the accumulation of toxic ions, and increasing K^+^ uptake. In addition, elevated levels of CAT, POD, and proline content.	[134]
	Foliar spray of Se-NPs (10 and 20 mg L^−1^)	Saline soils (0, 25, 50, and 75 mM NaCl)	Reducing lipid peroxidation and H_2_O_2_ content, by enhancing activities of antioxidant enzymes like SOD and POD. Enhanced levels of organic acids and sugars in the fruits	[135]
Tomato *Solanum lycopersicum* L.	Cu NPs 10 mg of Cu NPs absorbed on 1 g of CS–PVA hydrogel	100 mM NaCl in the nutrient solution	Increased the growth, yield, SOD, GSH, and GPX activity in leaves. Also, total phenol content increased in leaves, and lycopene and vitamin C content increased in fruits. ABTS increased, whereas DPPH decreased in leaves and fruits.	[136]
Cucumber *Cucumis sativus*	After 3 weeks of normal growth, the leaves were sprayed with Mn_3_O_4_ NPs suspensions (0, 20, or 100 mg L^−1^) twice daily, around 3.57 mL once/ plant.	0.3% NaCl	Mn_3_O_4_ NPs, at a concentration of 1 mg per plant, effectively reduced oxidative stress in cucumber plants, enabling them to preserve their biomass. Also, it increased the levels of endogenous antioxidants by upregulating precursors and downstream products in the shikimate and phenylpropanoid pathways.	[137]

**Table 3:** Nanomaterials and their effects on horticulture crops under biotic stress conditions.

Horticulture Crop Species	Nanomaterials	Type of Disease	Effects	References
Mango *Mangifera indica* L.	Chitosan nanoparticles (ChNPs) 0.25%, 0.5%, and 1%.	*Colletotrichum gloeosporioides*	The observed antifungal activity might be related to the interaction between the fungal cells and the ChNPs, where the surface area of the NPs makes it easier for them to be absorbed onto the cell surface. This changes the makeup of the cell and prevents vital nutrients from being accessible for growth.	[156]
	Sulfur nanoparticles (SNPs) 100, 300, and 500 ppm compared to micronized sulfur (500 ppm).	*Oidium mangiferae*	SNPs foliar spraying significantly reduced the disease incidence rate and severity index of powdery mildew in the Keitt mango cultivar. Besides an increase in the sulfur content of mango leaves, improved tree productivity, and affected the physical and chemical characteristics of fruits by reducing the H_2_O_2_ content.	[147]
Tomato *Solanum lycopersicum* L.	Silver and zinc oxide nanoparticles	*Tuta absoluta*	Causes oxidative stress by producing ROS, which damages proteins, lipids, and DNA in insect cells. This affects insects’ reproductive and developmental cycles, bolstering their potential as an efficient pest management method.	[157]
Cucumber *Cucumis sativus*	Silver nanoparticles 10, 30, 50, and 100 ppm	*Golovinomyces cichoracearum* or *Sphaerotheca fusca*	Ag NPs were applied 3~4 weeks before disease outbreaks; even 50 ppm of Ag NPs can effectively inhibit powdery mildew.	[158]

## Data Availability

Data are contained within the article.
